# Supplementary material for: Causal Relationship between Aging and Anorexia Nervosa: A White-Matter-Microstructure-Mediated Mendelian Randomization Analysis
Source: Biomedicines. 2024 Aug 16;12(8):1874. doi: 10.3390/biomedicines12081874 (PMC11351342; doi:10.3390/biomedicines12081874)
Supplement: Supplementary file 1 [file biomedicines-12-01874-s001.zip › Supplementary Figure/Figure S4/Singe SNP plot of MR analysis for TL to ubm-b-1662.pdf]

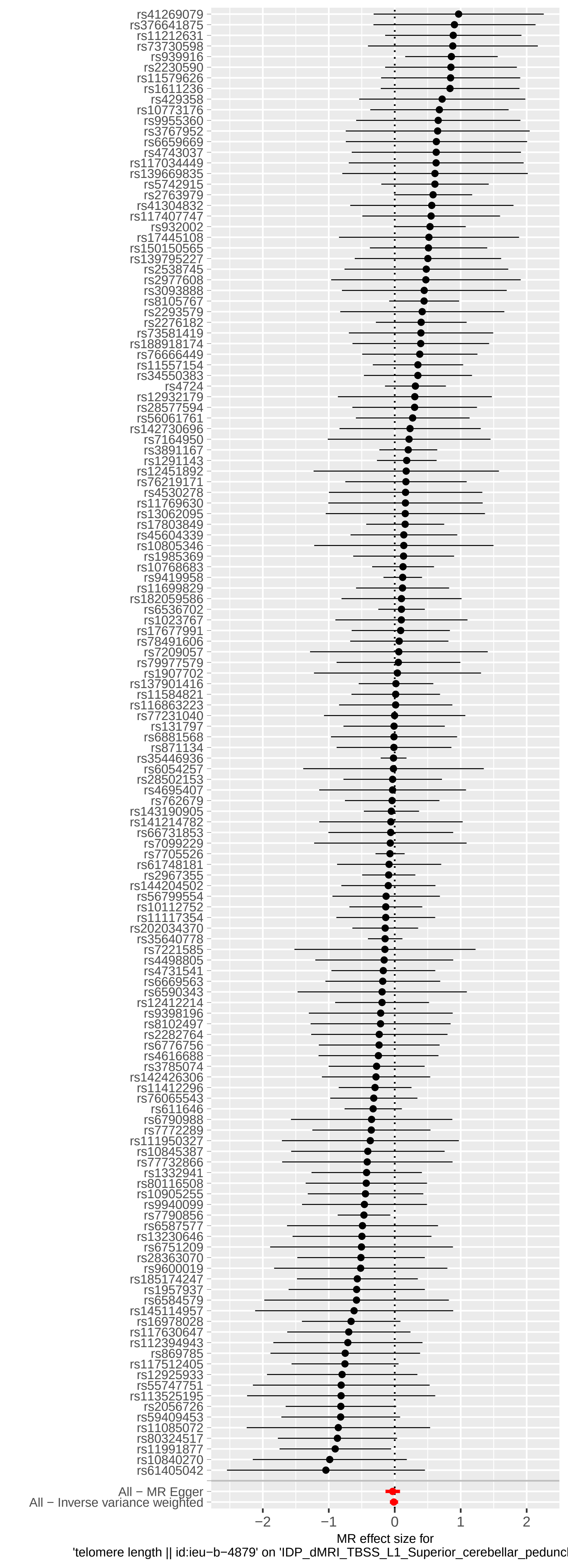
rs41269079
rs376641875
rs11212631
rs73730598
rs939916
rs2230590
rs11579626
rs1611236
rs429358
rs10773176
rs9955360
rs3767952
rs6659669
rs4743037
rs117034449
rs139669835
rs5742915
rs2763979
rs41304832
rs117407747
rs932002
rs17445108
rs150150565
rs139795227
rs2538745
rs2977608
rs3093888
rs8105767
rs2293579
rs2276182
rs73581419
rs188918174
rs76666449
rs11557154
rs34550383
rs4724
rs12932179
rs28577594
rs56061761
rs142730696
rs7164950
rs3891167
rs1291143
rs12451892
rs76219171
rs4530278
rs11769630
rs13062095
rs17803849
rs45604339
rs10805346
rs1985369
rs10768683
rs9419958
rs11699829
rs182059586
rs6536702
rs1023767
rs17677991
rs78491606
rs7209057
rs79977579
rs1907702
rs137901416
rs11584821
rs116863223
rs77231040
rs131797
rs6881568
rs871134
rs35446936
rs6054257
rs28502153
rs4695407
rs762679
rs143190905
rs141214782
rs66731853
rs7099229
rs7705526
rs61748181
rs2967355
rs144204502
rs56799554
rs10112752
rs11117354
rs202034370
rs35640778
rs7221585
rs4498805
rs4731541
rs6669563
rs6590343
rs12412214
rs9398196
rs8102497
rs2282764
rs6776756
rs4616688
rs3785074
rs142426306
rs11412296
rs76065543
rs611646
rs6790988
rs7772289
rs111950327
rs10845387
rs77732866
rs1332941
rs80116508
rs10905255
rs9940099
rs7790856
rs6587577
rs13230646
rs6751209
rs28363070
rs9600019
rs185174247
rs1957937
rs6584579
rs145114957
rs16978028
rs117630647
rs112394943
rs869785
rs117512405
rs12925933
rs55747751
rs113525195
rs2056726
rs59409453
rs11085072
rs80324517
rs11991877
rs10840270
rs61405042
All − MR Egger
All − Inverse variance weighted
−2
−1
0
1
2
MR effect size for
'telomere length || id:ieu−b−4879' on 'IDP_dMRI_TBSS_L1_Superior_cerebellar_pedunc
